# Giant thrombus in the left atrium after radiofrequency catheter ablation for atrial fibrillation: A case report

**DOI:** 10.1016/j.hrcr.2021.08.010

**Published:** 2021-09-02

**Authors:** Jun-ichi Noiri, Hiroki Konishi, Hiroki Matsuzoe, Shunsuke Sato, Takashi Azami, Kazuhiro Teramura

**Affiliations:** ∗ Department of Cardiology, Yodogawa Christian Hospital, Osaka, Japan; † Department of Cardiovascular Surgery, Yodogawa Christian Hospital, Osaka, Japan; ‡ Department of Pathology, Yodogawa Christian Hospital, Osaka, Japan

**Keywords:** Amaurosis fugax, Atrial fibrillation, Radiofrequency catheter ablation, Stroke, Thrombus

## Introduction

Radiofrequency catheter ablation (RFCA) is a standard treatment for atrial fibrillation (AF). However, RFCA is associated with perioperative problems, such as systemic embolization, and asymptomatic brain infarction is frequently detected by magnetic resonance imaging immediately after RFCA for AF.^1^^,^^2^ Most embolic adverse events associated with RFCA are presumably caused by microbubbles or thrombus formation.^3^ A previous in vivo study suggested that endothelial hyperthermic injury by RFCA induces thrombus formation.^4^ We report a rare case of a giant thrombus in the left atrium that induced brain infarction and amaurosis fugax 3 months after RFCA.

## Case report

An 81-year-old man with a history of myocardial infarction, diabetes mellitus, hypertension, and chronic kidney disease experienced multiple episodes of presyncope associated with paroxysmal AF. He underwent RFCA along with loop recorder implantation (Medtronic Reveal LINQ loop recorder; Medtronic Inc, Minneapolis, MN) to detect AF recurrence at another hospital.

The patient had a high risk of developing stroke owing to his advanced age, history of myocardial infarction, and the presence of comorbidities such as hypertension and diabetes mellitus (CHADS_2_ score, 3 points; CHA_2_DS_2_-VASc score, 5 points). In addition to aspirin (100 mg/day) for post–coronary stent implantation, an anticoagulant agent, rivaroxaban (10 mg/day), was prescribed in the perioperative period of RFCA. This is an appropriate dose for Japanese patients.^5^ The rivaroxaban was skipped once immediately before the catheter ablation procedure. Unfractionated heparin was given intravenously during the ablation procedure to sustain an activated clotting times of >300 seconds. The Japanese guidelines recommend this anticoagulation strategy before and during AF ablation.^6^ The outpatient doctor switched to apixaban (5 mg/day), which is also an acceptable dose in Japan for patients with renal function impairment.^7^ In high-risk patients (CHADS_2_ score ≥ 2), continuation of anticoagulation for as long as possible is recommended^6^; accordingly, we prescribed the patient direct oral anticoagulants.

**Figure 1:**
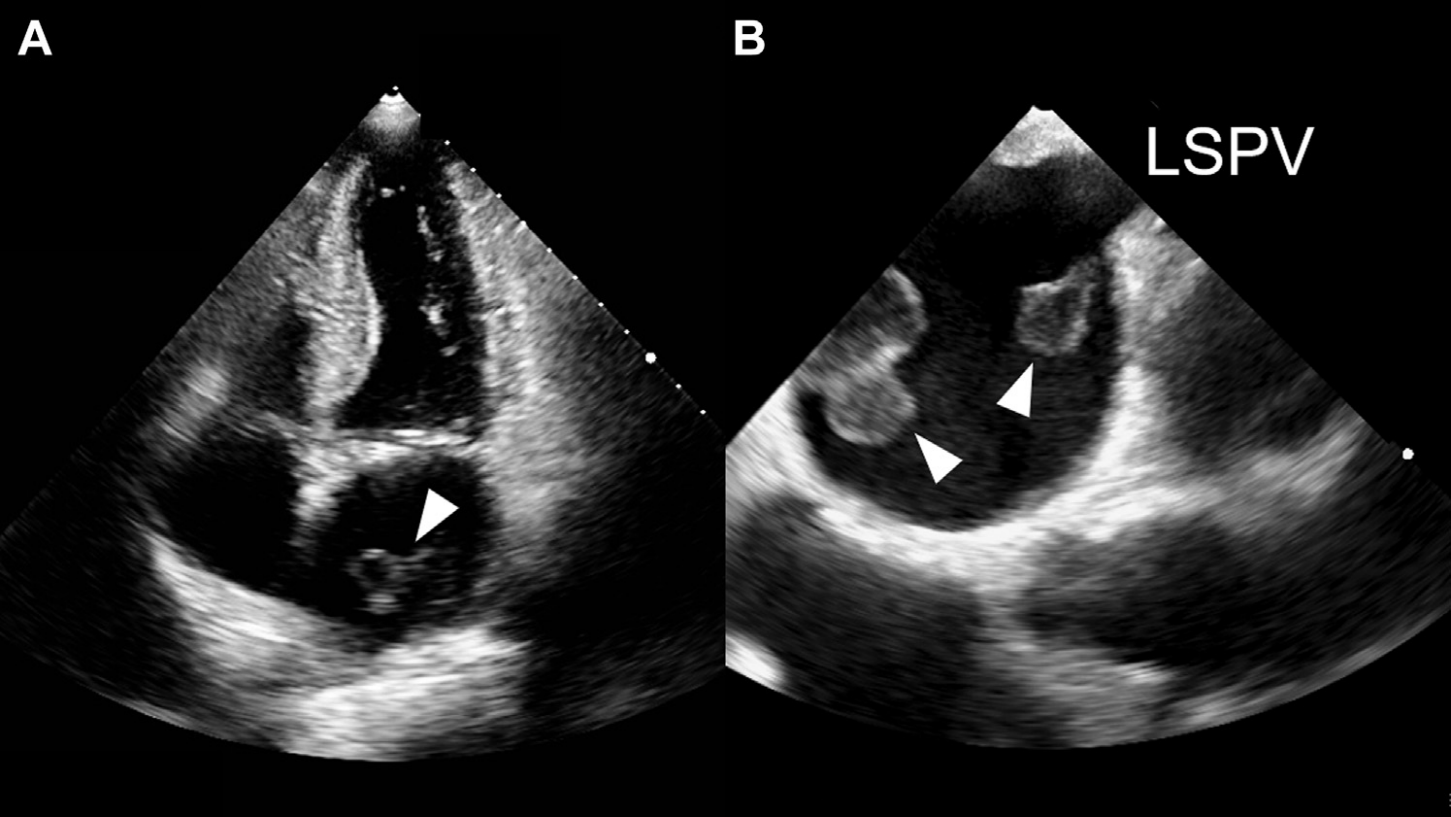
Giant thrombi in the left atrium visualized by transthoracic and transesophageal echocardiography. **A:** Transthoracic echocardiography detected large, abnormal structures with beaded appearance in the left atrium (*arrowhead*). The left atrium was dilated, but the other findings were not significant. **B:** Transesophageal echocardiography revealed de novo pedunculated giant mobile masses, which were attached to the ostia of the left superior and right inferior pulmonary veins (*arrowhead*). The surfaces of these masses were partially collapsed. Thrombus in the left atrial appendage and spontaneous echo contrast were not detected. LSPV = left superior pulmonary vein.

**Figure 2:**
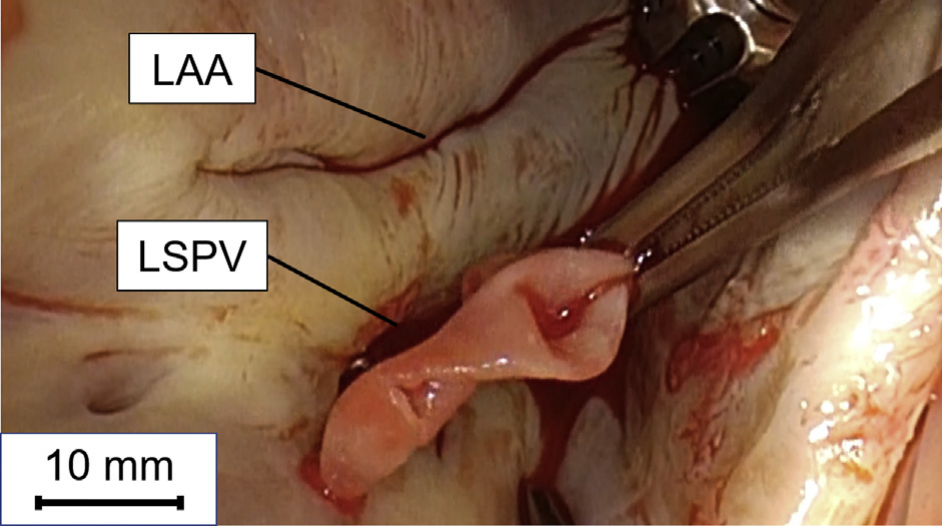
Intraoperative images of the thrombus attached to the ostium of the left superior pulmonary vein (LSPV). The intraoperative photograph shows a cystic mass in the left atrium attached to the ostium of the LSPV. The other mass was observed near to the right inferior pulmonary vein, as transesophageal echocardiography was performed before surgery. These adhesion sites corresponded to the ablation scar. LAA = left atrial appendage.

Exactly 3 months after RFCA, he presented to our hospital for blindness in the left eye, which persisted for approximately 30 minutes. Ophthalmoscopy did not reveal any abnormal findings. Visual impairment resolved at the time of admission. Although no irreversible neurological disorder could be detected, magnetic resonance imaging revealed signs of acute infarction of the left parietal lobe, supplied by the posterior cerebral artery arising from the vertebral artery. Magnetic resonance angiography revealed stenosis of the left carotid artery, but no evidence of stenosis of the left vertebral artery, suggesting that the thrombus at that stenosis may have caused amaurosis fugax. Magnetic resonance angiography could not explain the distribution of the infarction lesions. On transthoracic echocardiography, the left atrium was dilated, and the left ventricle function and valve were normal (left atrial dimension 49 mm, left atrial volume 80.9 mL, left atrial volume index 48.5 mL/m^2^). Large, abnormal structures with beaded appearance were detected in the left atrium (Figure 1A). Transesophageal echocardiography revealed 2 de novo pedunculated giant mobile masses, which were attached to the ostia of the left superior and right inferior pulmonary veins (Figure 1B). The adhesion site of the mass corresponded to the location of ablative lesions. The partially collapsed surface of these masses was considered an embolic source. Emergency surgical removal of the left atrial mass combined with a maze procedure was performed to prevent further systemic embolism. Corresponding to the findings of echocardiography, each mass was attached to the ablative lesion beside the ostia of the left superior and right inferior pulmonary veins (Figure 2).

**Figure 3:**
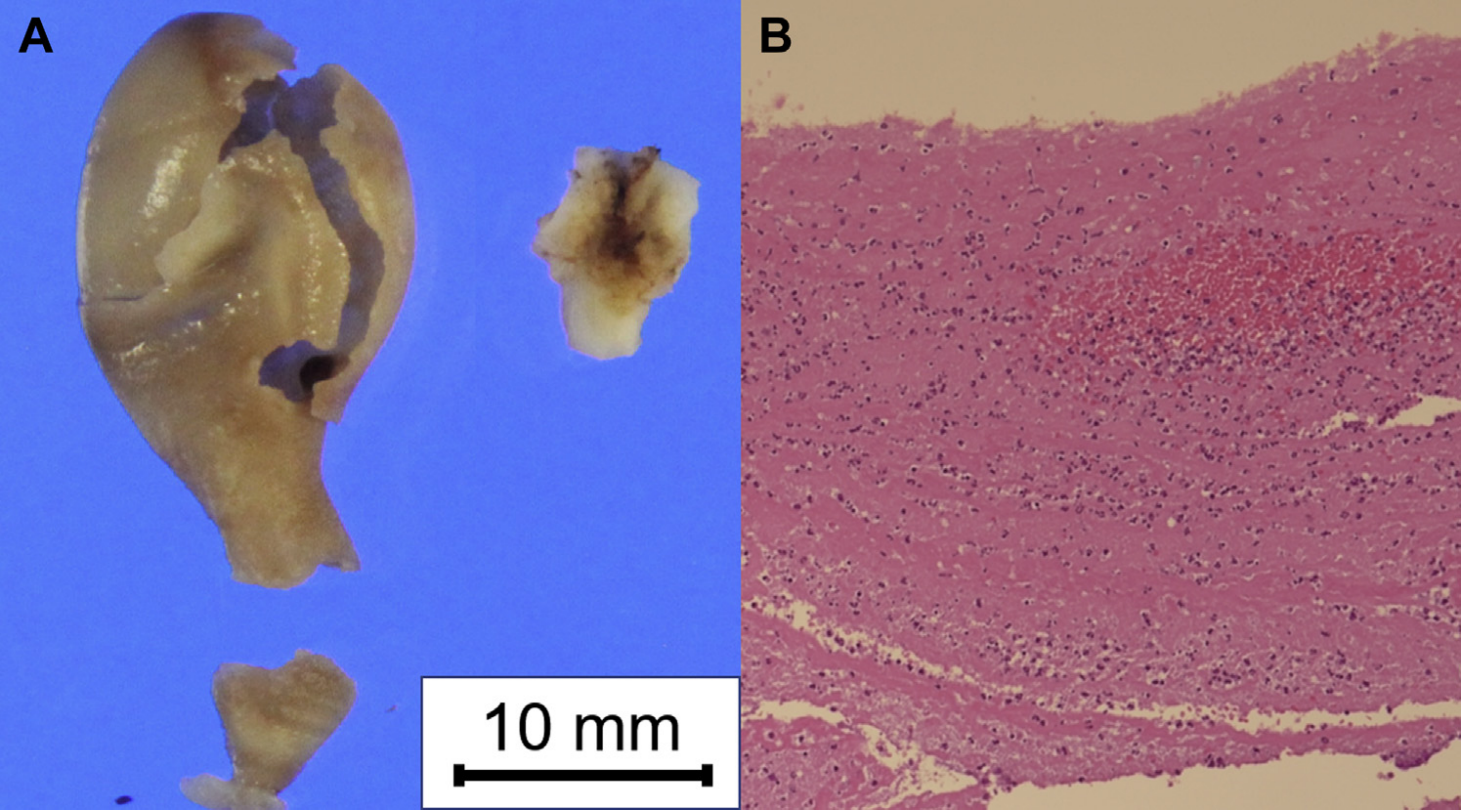
Macroscopic and microscopic pathology images. **A:** Pathologic assessment revealed a cystic mass of 21 × 12 mm in size, which was attached to the left atrial roof near to the left superior pulmonary vein. **B:** Hematoxylin–eosin staining showed mixed thrombi consisting of fibrin and platelets, including infiltration of numerous neutrophils and histiocytes. No neoplastic cells or bacteria were observed.

Pathologic assessment showed cystic masses of 21 × 12 mm and 23 × 12 mm in size, which were attached to the left atrial roof near to the left superior and right inferior pulmonary veins (Figure 3A). Microscopic evaluation revealed mixed thrombi consisting of fibrin and platelets, including infiltration of numerous neutrophils and histiocytes, and there were no neoplastic cells or bacteria (Figure 3B). A portion of the cystic mass attached to the pulmonary vein included fibrofatty connective tissue.

The patient was confirmed to have 2 other risk factors for thromboembolism after the surgery. First, he was diagnosed with essential thrombocythemia. His platelet count increased 1 week after cardiac surgery, and peripheral blood testing was positive for the *JAK2* V617F mutation. Second, 1 year after the cardiac surgery, he was found to have a malignant cancer that had metastasized to multiple organs. He had a medical history of early gastric cancer that had been managed by curative distal gastrectomy 16 years prior, and prostate cancer that was treated with radiotherapy 4 years prior. Planned follow-up for cancer was performed. One year after intracardiac thrombectomy, pleural and pericardial effusions induced by adenocarcinoma were detected. Positron emission tomography–computed tomography revealed recurrent gastric cancer with peritoneal dissemination. Furthermore, microscopic examination of the urine indicated urothelial carcinoma.

After surgical treatment, the prothrombin time–international normalized ratio was maintained within the therapeutic range (2.0–3.0; target 2.5) using warfarin, which was chosen because the thrombus in the left atrium had formed under anticoagulation by direct oral anticoagulants, and we believed that more aggressive and monitored anticoagulation was needed. Five months after surgical treatment, transesophageal echocardiography indicated no recurrence of the thrombosis in the left atrium. We continued anticoagulant treatment for 1 year until his malignant findings became apparent.

## Discussion

This case highlights that RFCA carries a risk of thrombus formation, leading to a periprocedural complication of systemic embolization. To the best of our knowledge, there are few reported cases of giant thrombus in the left atrium associated with RFCA. In a case similar to ours, a giant left atrial mass was found in a 71-year-old man who had undergone RFCA for AF 3 months before.^8^ As the mass did not shrink in size despite anticoagulation treatment, it was surgically removed. Pathologic examination confirmed that it was a thrombus. This case also suggests that the risk of left atrial clot formation increases following RFCA despite anticoagulation therapy.

In our case, the patient was at high risk of thromboembolism. In addition to the high CHADS_2_ score, he was found to have essential thrombocythemia and carcinoma after the cardiac surgery. Even though these diseases were undiagnosed at the time of ablation, they may have promoted thrombus formation after the procedure. Thrombus formation in cancer patients is associated with proliferation and metastasis.^9^ However, the patient received adequate anticoagulation treatment, and the implanted loop recorder did not detect an episode of AF recurrence. Furthermore, RFCA was conducted using an irrigated catheter, ThermoCool SmartTouch Surround Flow (Biosense Webster, Diamond Bar, CA), with contact force and ablation index navigation. There was no occurrence of steam pop. Despite these conditions, thrombi formed on the ablative lesion induced by RFCA. This suggests that endothelial injury by RFCA may be a trigger for exacerbated thrombosis.

We report a rare case of brain infarction and amaurosis fugax due to a giant thrombus in the left atrium after RFCA for AF. In this case, essential thrombocythemia and cancer, as well as endothelial damage with RFCA, may have promoted thrombosis. It is important to recognize this complication when patients who undergo RFCA show signs of thromboembolism, particularly in cases at high risk of thrombosis.

**Key Teaching Points**

• This report summarizes a rare case of brain infarction and amaurosis fugax due to a giant left atrial thrombus 3 months after radiofrequency catheter ablation (RFCA) for atrial fibrillation (AF) in an 81-year-old man.
• Cardiac thrombectomy revealed that the thrombus was on the ablation line, suggesting that the endothelial damage with RFCA may have promoted thrombosis.
• This patient had essential thrombocythemia and advanced malignant cancer, both of which are risk factors for thromboembolism. This case suggests the importance of evaluation for the risk of thromboembolism after RFCA, even in cases with adequate anticoagulation or nonrecurrence of AF.
